# *Helianthus Annuus* L. ameliorates atherosclerosis-induced myocardial infarction via inhibiting inflammation and oxidative stress

**DOI:** 10.3389/fcvm.2025.1744283

**Published:** 2026-01-06

**Authors:** Mengjue Li, Jianbing Wang, Lina Chen, Shenqing Cui, Bowen Jiang, Haoran Sun, Yunxia Zhu

**Affiliations:** 1 Department of Biochemistry and Molecular Biology, Nanjing Medical University, Nanjing, Jiangsu, China; 2 Department of Cardiology, North China Petroleum Administration Bureau General Hospital, Renqiu, Hebei, China; 3 Department of Cardiology, Shaoxing Central Hospital Medical Alliance General Hospital, Shaoxing, Zhejiang, China

**Keywords:** atherosclerosis, *Helianthus Annuus* L., inflammation, myocardial infarction, oxidative stress

## Abstract

**Background:**

The majority of myocardial infarction (MI) cases occur due to plaque rupture in the context of atherosclerosis (AS). This study aimed to elucidate the mechanisms underlying the effect of *Helianthus Annuus* L. (HAL) on MI in the setting of AS.

**Methods:**

Apolipoprotein E-deficient mice were divided into four groups of 11. Mice in the Control and MI groups were fed a normal diet for 24-weeks, while mice in the AS + MI group were fed a high-fat diet (HFD) and mice in the AS + MI + HAL group were fed HFD supplemented with 5% HAL powder. After 23 weeks, the mice in the MI, AS + MI and AS + MI + HAL groups underwent coronary artery ligation to induce MI. A week post-ligation, 6 surviving mice were randomly selected from each group for the subsequent experiments, echocardiography was performed, followed by analysis of aortic plaque and myocardial tissue to explore potential mechanisms.

**Results:**

HAL intervention attenuated cardiac remodeling and dysfunction induced by MI, reduced inflammatory cell infiltration and fibrosis in the myocardium, and consequently improved cardiac function. HAL alleviated the inflammatory response by reducing serum concentrations and myocardial expression of interleukin-1β, interleukin-6 and tumor necrosis factor-α. Furthermore, Western blotting analysis demonstrated that HAL reduced nuclear factor-κB (NF-κB) expression. HAL also enhanced superoxide dismutase and glutathione peroxidase levels while suppressing malondialdehyde and myeloperoxidase levels in cardiac infarction tissue.

**Conclusion:**

Our data indicated that HAL attenuates cardiac remodeling by inhibiting inflammation and reducing oxidative stress. These findings provide novel insights into the effects and mechanisms of HAL in the context of MI.

## Introduction

Myocardial infarction (MI) is one of the most prevalent cardiovascular disease, characterized by acute myocardial injury resulting from prolonged myocardial ischemia (1). It is associated with elevated mortality, as well as a high incidence of arrhythmias and heart failure. Coronary thrombosis superimposed on atherosclerotic plaques remains the hallmark and principal therapeutic target in MI (2). Specifically, MI resulting from the rupture of cholesterol-rich atherosclerotic plaques is strongly linked to cardiac death (3). As ischemic heart disease continues to be the predominant cause of heart failure, understanding and intervening in the post-infarction pathological processes have become urgent priorities in cardiovascular research (4).

A key pathological feature after MI is ventricular remodeling, which involves structural and functional changes in the heart resulting from responses of cardiomyocytes, stromal cells, and the extracellular matrix to ischemic injury (5). The inflammatory response, while essential for clearing cellular debris, can become maladaptive when excessive or prolonged, exacerbating tissue damage and dysfunction. Coupled with oxidative stress, this dysregulated inflammation promotes extracellular matrix degradation, cardiomyocyte apoptosis, wall thinning, chamber dilation, and fibrotic scar formation—all contributing to mechanical deficit and progressive heart failure (6). Thus, controlling inflammation and oxidative damage has emerged as a promising therapeutic strategy to preserve cardiac function post-MI (7).

Inflammation orchestrates the constellation of pathophysiological processes—including injury, repair, and remodeling—in the infarcted heart, and has thus emerged as a viable therapeutic avenue for improving post-MI prognosis (8). Despite encouraging preclinical results, many anti-inflammatory and antioxidant agents have shown limited efficacy in clinical trials, highlighting the need for safer and more effective treatment alternatives. Ischemic heart disease treatments and preventions by using plant extract and its phytochemical constituents have achieved considerable attention globally due to its cardioprotective effects (9). Nutraceuticals derived from natural products offer a compelling avenue, owing to their multi-target effects and favorable safety profiles (10). The flower heads of Helianthus annuus L. (HAL) are abundant in bioactive flavonoids and polysaccharides. Previous work by our group has demonstrated their potent anti-inflammatory and antioxidant activities (11). The results from our study have demonstrated that HAL alleviated atherosclerotic plaque formation by inhibiting inflammation and restraining oxidative stress in AS mouse model.

Based on this rationale, the present study aimed to evaluate the cardioprotective effects of HAL in a mouse model combining atherosclerosis and myocardial infarction. Through comprehensive physiological, biochemical, and molecular analyses, we aimed to elucidate whether HAL attenuates pathological remodeling, modulates inflammatory and oxidative pathways, and ultimately improves cardiac function after MI.

## Materials and methods

### Animals

All experimental protocols were conducted in accordance with the Terrestrial Animal Health Code (24) and approved by the Animal Ethics and Welfare Committee of the Institute of Radiology, Chinese Academy of Medical Sciences (Approval No. IRM-DWLL-2019004). Female apolipoprotein E-deficient (Apoe^−/−^) mice on a C57BL background were obtained from the Model Animal Research Center of Nanjing University. Upon arrival at our animal facility at 7 weeks of age, mice were housed under specific pathogen-free (SPF) conditions. Standard husbandry practices included regular replenishment of feed, water, and bedding. Mice were maintained on a 12 h light/dark cycle with *ad libitum* access to food and water. Environmental conditions were maintained at 22–24  °C and 45%–55% relative humidity. Following a one-week acclimatization period, the experimental procedures commenced.

### Preparation of diet

HAL heads were collected from saline-alkali fields in Jilin Province, China. After seed removal, the heads were dried and coarsely ground to yield Helianthus annuus head powder (HAL). The powder was stored in airtight, light-proof containers at 0–4  °C until use. The composition of the HAL head powder included a mixture of neutral sugars (such as D-xylanose) (59.3%), α-cellulose (52%), pectin (27.5%) and lignin (12.3%) (12). Acetone extracts of HAL were fractionated using chromatographic techniques as previously described (13). This process isolated eleven diterpenoid compounds, whose structures were elucidated through spectral analysis and comparison with published data.

The high-fat diet (HFD) contained 40% fat by weight. The HAL-supplemented diet (HAL-HFD) comprised 5% Helianthus annuus head powder (HAL) blended into the HFD base. Protein and fat content were identical between HFD and HAL-HFD formulations. All experimental diets were stored in light-protected containers at 0–4  °C until use.

### Myocardial infarction induction in atherosclerotic Apoe^−/−^ mice

The mice were randomly divided into four groups of 11. The Control and MI groups were given normal diet. The AS + MI group was given HFD. The AS + MI + HAL group was given HFD mixed with 5% HAL powder. All mice were housed under SPF conditions and maintained on their respective diets for 24 weeks. After 23 weeks of feeding, mice were fasted for 12 h prior to MI modeling. In the induction room, anesthesia was induced using 2%–3% isoflurane delivered via a small animal anesthesia machine. Upon successful anesthesia, mice in the MI, AS + MI, and AS + MI + HAL groups were secured in the supine position on the operating table using adhesive tape applied to the abdomen. The light source of the dissection microscope was activated, and a small animal ventilator was connected to provide respiratory support via an oral interface. Ventilator parameters were set as follows: respiratory rate 80 breaths/min, tidal volume 4 ml, and inspiration-to-expiration ratio (I: E) of 1:1. Myocardial infarction was induced using a novel minimally invasive technique, as previously described (14), which involved ligating the mid-left anterior descending (LAD) coronary artery. This approach achieves high reproducibility and offers significant animal welfare benefits by avoiding thoracotomy. Post-surgery, mice were monitored closely and bedding was changed daily to minimize the risk of wound infection. One week after MI, 6 surviving mice were randomly selected from each group for the subsequent experiments. Echocardiography (using the VEVO2100 system) was performed to assess cardiac function.

### Determination of heart failure biomarkers

Following echocardiography, mice were anesthetized and subjected to terminal blood collection via the orbital plexus. Serum was isolated by centrifugation at 3,000 rpm for 10 min at 4  °C. Serum concentrations of N-terminal pro-B-type natriuretic peptide (NT-proBNP) were quantified using commercial ELISA kits (BlueGene Biotech, Shanghai, China) according to the manufacturer's protocol, as previously described.

### Histopathological analysis of atherosclerotic lesions and myocardial infarction

Atherosclerotic lesions were assessed in aortic arches and thoracic aortae using Oil Red O (ORO) staining. Lipid deposition was quantified through computer-assisted morphometric analysis of ORO-positive areas. The stained plaque area was quantified as a percentage of the total aortic sinus area using ImageJ software (NIH).

Left ventricles were sectioned longitudinally (apex-to-base orientation) at 6 μm thickness for comprehensive assessment of cellular morphology (H&E staining) and interstitial collagen deposition (Masson staining). The inflammatory and fibrotic area (stained blue) was quantified as a percentage of the total myocardial area in the field using ImageJ software's color threshold analysis tool.

### Quantification of Serum inflammatory factors

Serum concentrations of interleukin (IL)-1β, IL-6, and tumor necrosis factor (TNF)-*α* were quantified using enzyme-linked immunosorbent assay (ELISA) kits (BlueGene Biotech, Shanghai, China). All assays were performed in duplicate according to the manufacturer's protocol with incubation at 37°C, as previously described (15)

### Quantitative analysis of cardiac inflammatory gene expression via RT-PCR

Cardiac tissues from the infarct zone were harvested, snap-frozen in liquid nitrogen, and stored at −80 °C until analysis. For RNA extraction, 50 mg of apical myocardial tissue was homogenized in 450 μL ice-cold PBS using a sonicator (30% amplitude, 10 s pulses ×3). The homogenate was centrifuged at 3,000 rpm for 10 min at 4 °C to obtain a 10% (w/v) tissue supernatant.

Total RNA was isolated using the RNA prep Pure Tissue Kit (Tiangen Biotech, Beijing, China) according to the manufacturer's protocol. RNA integrity was verified by A_260_/A_280_ ratios (1.8–2.0). First-strand cDNA was synthesized from 1 μg total RNA using the FastKing RT Kit (Tiangen). RT-qPCR amplification was performed in triplicate on a Bio-Rad iQ5 system (Hercules, CA, USA). Gene expression of IL-1β, IL-6, and TNF-α was quantified using the 2^−*ΔΔ*CT^ method with β-actin as the endogenous control, as previously validated (16). The sequences of the primers were listed in Table 1.

**Table 1:** Primer sequences for mouse target genes.

Genes	Primer sequence (5'–3')
IL-6	Forward ACA AAG CCA GAG TCC TTC AGA
	Reverse TGT GAC TCC AGC TTA TCT CTT GG
IL-1β	Forward GTC CAA TTC GTT GTG GGC AT
	Reverse CTC CCC TGG GAC ACA TCA AG
TNF-α	Forwad ACT CCA TCG GGG TTA ATG CT
	Reverse GAC TCA GCA TCA CCG TAGT TT
β-Actin	Forwad GGC TGT ATT CCC CTC CAT CG
	Reverse CCA GTT GGT AAC AAT GCC ATG T

### Expression of nuclear factor-κB (NF-κB) in myocardial infarction tissue

Following the preparation of a 10% homogenate from infarcted myocardial tissue, NF-κB expression was assessed by Western blotting.

The tissues were lysed using ice-cold RIPA buffer (Thermo Fisher Scientific) containing 0.5 mM EDTA and Halt™ protease/phosphatase inhibitor cocktail (Thermo Fisher Scientific). The lysates were rotated at 4 °C for 15–30 min and then centrifuged at maximum speed for 15 min to obtain whole-cell extracts. Protein concentration was determined using the BCA protein assay (Takara). Thirty micrograms of total protein per sample were separated on 4%–12% NuPAGE™ Tris-Bis gradient gels (Thermo Fisher Scientific) via SDS-PAGE and subsequently transferred to PVDF membranes (Millipore Sigma). The membranes were blocked with 5% non-fat milk. GAPDH was used as a loading control. Primary antibodies were detected with HRP-conjugated secondary antibodies (Sigma-Aldrich), and signals were visualized using chemiluminescent substrate (PerkinElmer ECL). Protein band quantification was performed using ImageJ software (NIH Image). Key reagents and antibodies are listed in Table 2.

**Table 2:** Antibodies used for western blot analysis.

Antigen	Species	Manufacturer and Cat. #	Dilution
P-p65 (Ser536)	Rabbit	CST, America, #3033	1:1000
p65	Rabbit	CST, America, #8242	1:1000

### Assessment of cardiac oxidative stress markers

A 10% tissue homogenate of the heart was prepared. Oxidative stress parameters were quantified using commercial assay kits (Nanjing Jiancheng Bioengineering Institute, China) according to standardized protocols. Malondialdehyde (MDA) was quantified via thiobarbituric acid (TBA) assay based on MDA-TBA adduct formation at 532 nm (Kit No. LCP2299). Myeloperoxidase (MPO) activity was measured by colorimetric detection of tetramethylbenzidine oxidation at 450 nm (Kit No. LCP2110). Superoxide dismutase (SOD) activity was determined using WST-8 reduction method monitoring formazan formation at 450 nm (Kit No. LCP1030). Glutathione peroxidase (GSH-Px) activity assessed through NADPH oxidation kinetics at 340 nm (Kit No. LCP2310). All assays were performed in triplicate with tissue homogenates centrifuged at 12,000 rpm for 15 min at 4 °C prior to analysis. Data normalization was based on total protein concentration determined by BCA assay.

### Statistics

Statistical analyses were performed using SPSS 25.0 (IBM Corp., Armonk, NY, USA) and GraphPad Prism 8.0 (GraphPad Software, San Diego, CA, USA). Continuous variables are presented as mean ± standard deviation from at least three independent experiments. Normality of distribution was verified by the Shapiro–Wilk test. Between-group differences were analyzed by one-way analysis of variance (ANOVA) with Tukey's *post hoc* test for multiple comparisons. Statistical significance was defined as *p* < 0.05 (two-tailed). Dose-response curves were fitted using nonlinear regression analysis in GraphPad Prism.

## Results

### Comparison of left ventricular remodeling and function after MI

**Figure 1:**
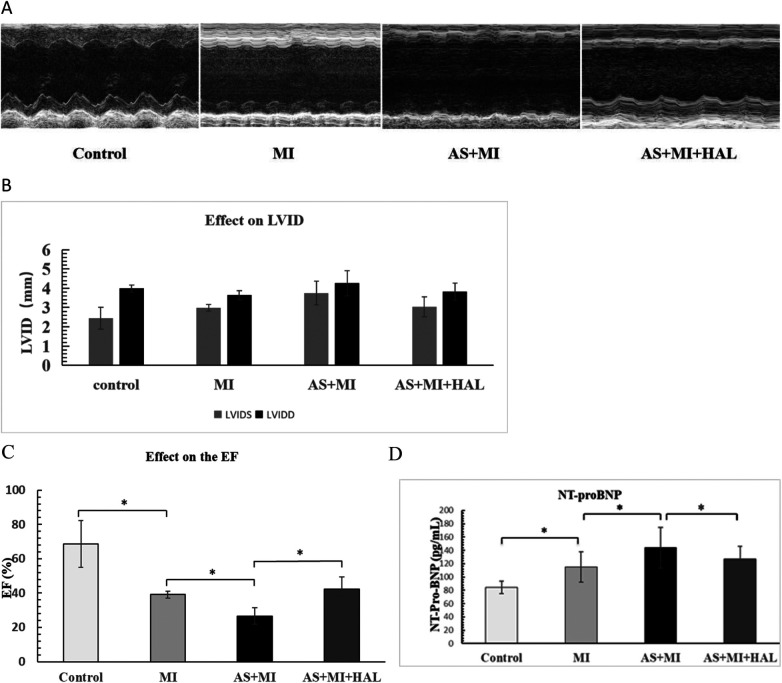
**(A)** AS significantly aggravated cardiac remodeling and dysfunction after MI, and HAL improved cardiac function. M-mode ultrasound imaging of left-ventricular structure. **(B)** Left ventricular internal dimensions post-MI. **(C)** Changes in ejection fraction (EF). **(D)** Serum NT-proBNP levels. Values are mean ± SEM (*n* = 6). *p < 0.05.

Left ventricular structure and systolic function at one week after LAD ligation in mice (Figure 1). Echocardiographic assessment under baseline conditions revealed that left ventricular internal dimensions were significantly increased in mice following MI. Specifically, both end-systolic diameter (LVIDS: 3.74 mm vs. 2.99 mm) and end-diastolic diameter (LVIDD: 4.26 mm vs. 3.63 mm) were greater in the AS + MI group compared to the MI group (Figure 1B). Intervention with HAL markedly attenuated this remodeling. The AS + MI + HAL group exhibited reduced LVIDS (3.05 mm vs. 3.75 mm) and LVIDD (3.82 mm vs. 4.26 mm) relative to the AS + MI group (Figure 1B). Additionally, the decline in ejection fraction (EF) following MI was significantly ameliorated by HAL treatment. The AS + MI + HAL group maintained an EF of 42%, compared to 26% in the AS + MI group (Figure 1C).

Serum levels of the heart failure biomarker NT-proBNP were shown in Figure 1D. Both the MI and AS + MI groups exhibited significantly elevated NT-proBNP concentrations compared to the control group (*p* < 0.05). Furthermore, the AS + MI group showed a further increase in NT-proBNP compared to the MI group (*p* < 0.05). In contrast, HAL treatment significantly reduced NT-proBNP levels in the AS + MI + HAL group relative to the AS + MI group (*p* < 0.05).

### Post-MI mortality

**Figure 2:**
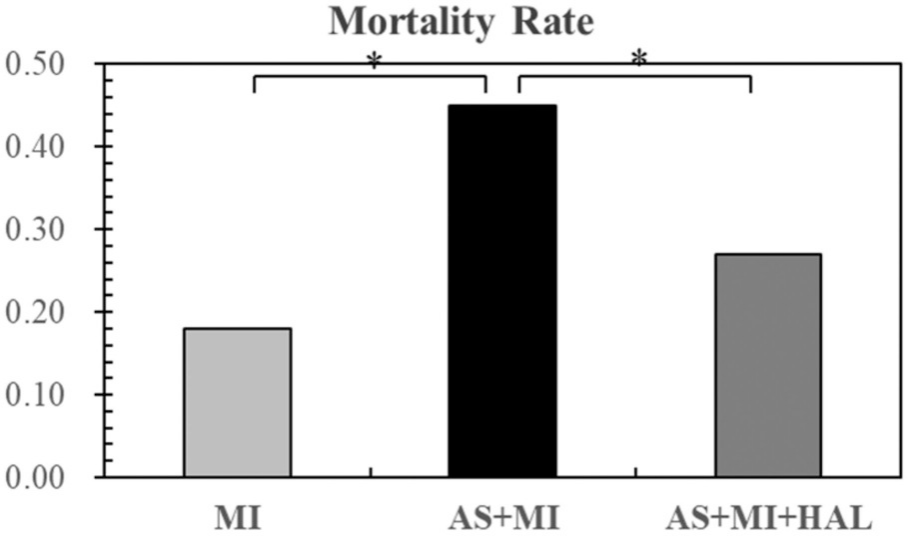
AS increased post-MI mortality risk, and HAL treatment reduced mortality. Values are mean ± SEM (*n* = 11). *p < 0.05.

Figure 2 displayed the mortality rate within one week after MI. AS significantly increased the risk of mortality following MI, whereas HAL treatment effectively reduced the mortality rate.

### Atherosclerotic plaque formation and lipid content

**Figure 3:**
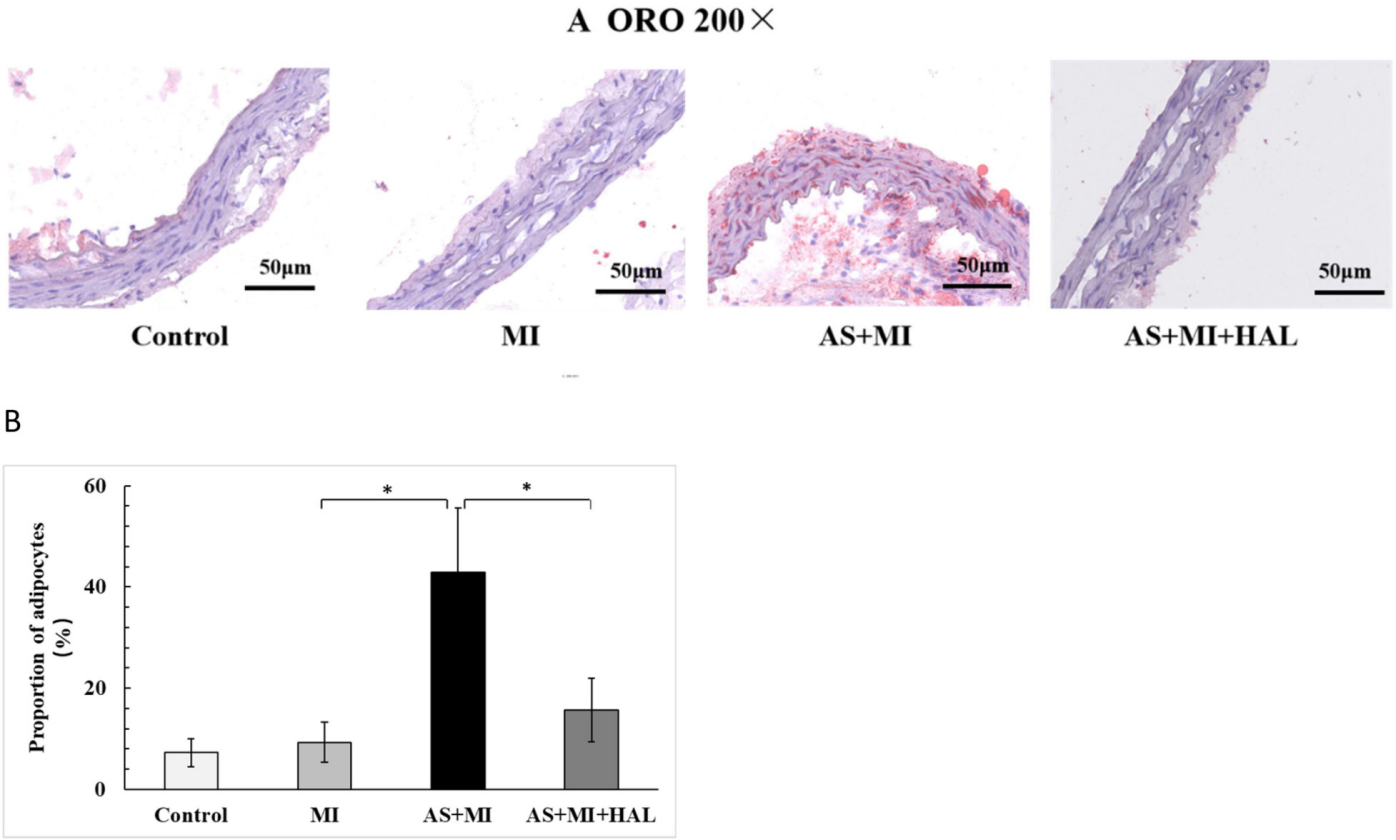
HFD increased lipid accumulation in atherosclerotic plaques, and HALtreatment reduced it. Values are mean ± SEM (*n* = 6). *p < 0.05.

In our previous experiments, lipid-laden plaques were observed throughout the aortic tissue in the AS model group following 24 weeks of HFD feeding (17). The same result was obtained in this experiment. To evaluate the lipid content within atherosclerotic plaques, ORO staining was performed (Figure 3). The HFD markedly promoted lipid accumulation, as evidenced by an increased percentage of adipocyte area. In contrast, HAL treatment led to a substantial reduction in lipid deposition, indicating attenuation of atherosclerotic plaque formation.

### Myocardial tissue structure in mice

**Figure 4:**
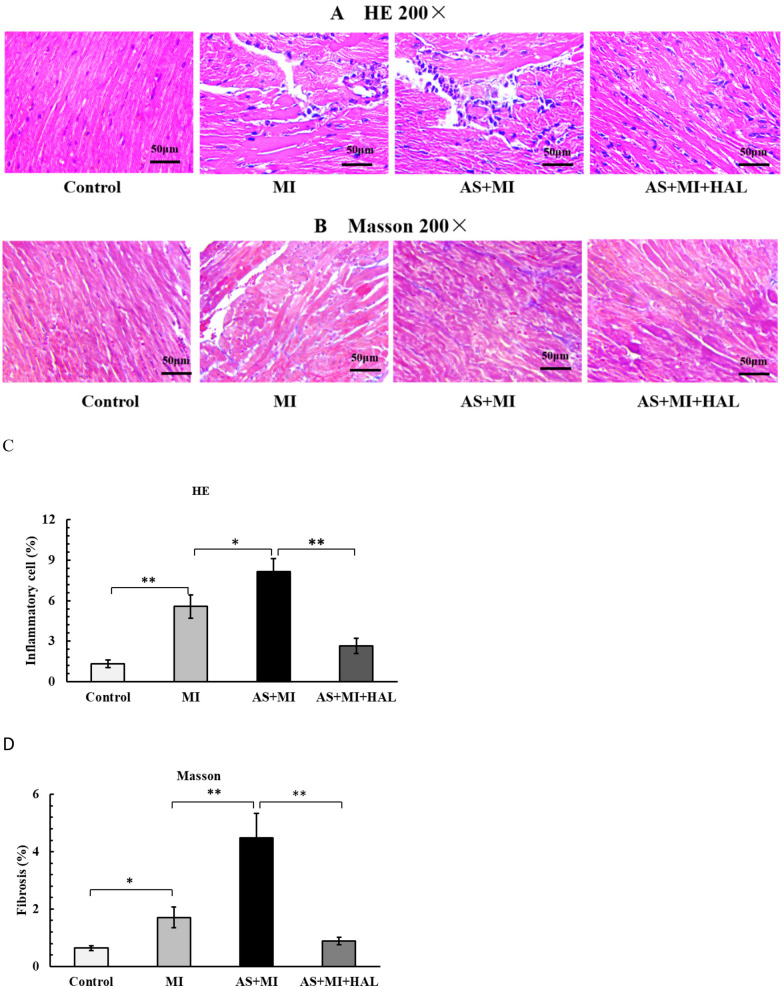
**(A,C)** H&E staining (200×) showed the myocardial inflammatory cell infiltration. **(B,D)** Masson staining (200×) showed the myocardial fiber tissue filling. Values are mean ± SEM (*n* = 6). *p < 0.05; **p < 0.01.

The myocardial tissue structure of mice was examined as shown in Figure 4. MI significantly increased inflammatory cell infiltration (*p* < 0.01) and myocardial fibrosis (*p* < 0.05). Furthermore, the combination of AS and MI resulted in a substantial enhancement in positive staining for H&E (Figures 4A,C) and ORO (Figures 4B,D), indicating aggravated damage to the heart muscle. In contrast, intervention with HAL significantly reduced the proportion of positive staining for both H&E and Masson trichrome compared to the AS + MI group (*p* < 0.01), suggesting alleviated myocardial injury.

### Impact of HAL on serum levels and cardiac expression of IL-1β, IL-6, and TNF-α following MI

**Figure 5:**
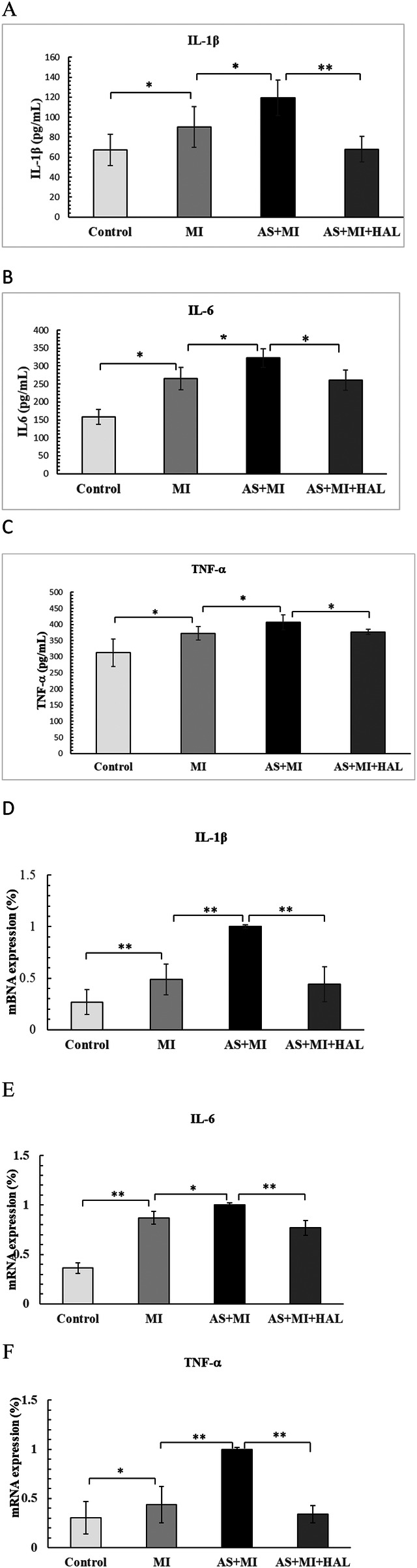
Effects of HAL on pro-inflammatory cytokines. Serum levels of IL-1β**(A)**, IL-6 **(B)** and TNF-α **(C)** Cardiac tissue expression of IL-1β **(D)**, IL-6 **(E)** and TNF-α **(F)** Data are mean ± SD (*n* = 6). *p < 0.05; **p < 0.01.

The serum concentrations and cardiac tissue expression levels of the pro-inflammatory cytokines IL-1β, IL-6, and TNF-α were presented in Figure 5. Compared with the control group, the MI group exhibited significantly elevated serum levels of IL-1β, IL-6, and TNF-α (*p* < 0.05). These cytokine levels were further increased in the AS + MI group compared to the MI group (*p* < 0.05). Treatment with HAL significantly reduced serum concentrations of IL-1β (*p* < 0.01), as well as IL-6 and TNF-α (*p* < 0.05), relative to the AS + MI group. A consistent trend was observed in cardiac tissue, where HAL treatment also markedly suppressed the expression of IL-1β, IL-6, and TNF-α (*p* < 0.01).

### Effect of HAL on the NF-κB inflammatory pathway in cardiac infarction tissue

**Figure 6:**
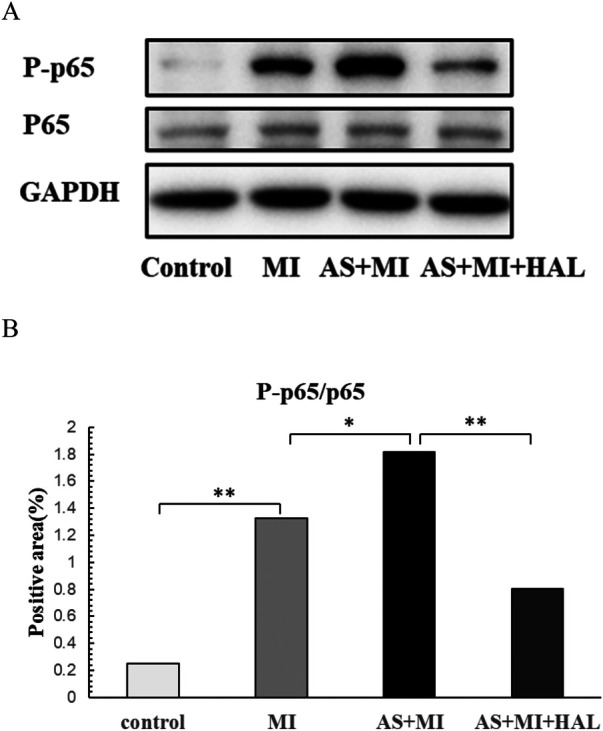
Effects of HAL on NF-*κ*B expression. WB results of P-p65 and P65**(A)**, Quantification of P-p65/P65 **(B)** Data are mean ± SD (*n* = 6). *p < 0.05; **p < 0.01.

The expression levels of phosphorylated p65 (P-p65) and total p65 in cardiac infarction tissue are shown in Figure 6. Compared with the control group, the expression of P-p65 was significantly increased in the MI group (*p* < 0.01). Furthermore, AS exacerbated the cardiac inflammatory response compared to the MI group, as indicated by a further elevation in P-p65 expression (*p* < 0.05). Treatment with HAL significantly reduced the expression of P-p65 compared to the AS + MI group (*p* < 0.01). In contrast, the expression of total P65 remained consistent across all groups without significant changes.

### Impact of HAL on oxidative stress markers in cardiac infarction tissue

**Figure 7:**
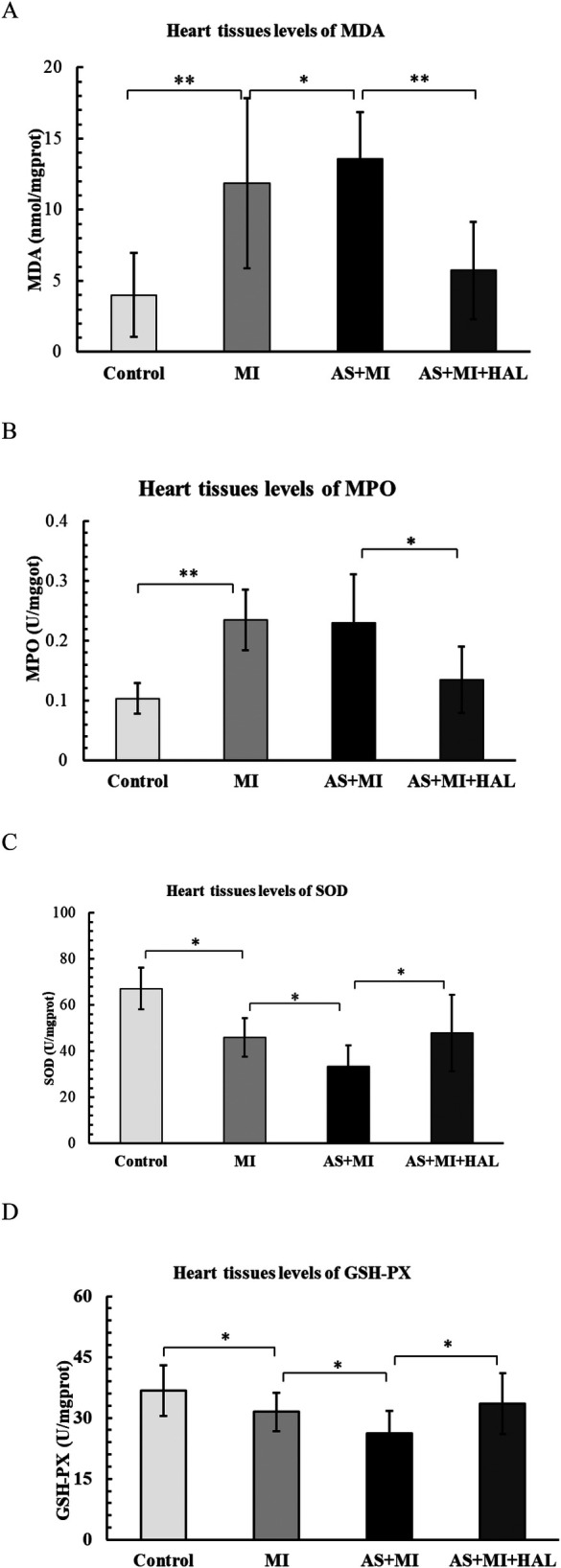
Effects of HAL on oxidative stress markers. Levels of MDA **(A)**, MPO **(B)**, SOD **(C)** and GSH-Px **(D)** in cardiac tissues. Data are mean ± SD (*n* = 6). *p < 0.05; **p < 0.01.

The levels of MDA, MPO, SOD, and GSH-Px in cardiac tissues were shown in Figure 7. Compared with the control group, the MI group exhibited a significant increase in the pro-oxidant markers MDA and MPO (*p* < 0.01), along with a significant decrease in the antioxidant enzymes SOD and GSH-Px (*p* < 0.05). AS further aggravated the oxidative stress response compared to the MI group. Treatment with HAL significantly reduced the levels of MDA (*p* < 0.01) and MPO (*p* < 0.05), while markedly increasing the activities of SOD and GSH-Px (*p* < 0.05) compared to the AS + MI group.

## Discussion

This study systematically evaluated the impact of HAL intervention on left ventricular remodeling and function following MI, particularly in the context of underlying AS. The principal findings demonstrate that HAL treatment effectively improved post-MI cardiac structure and function, attenuated maladaptive ventricular remodeling, reduced mortality, and significantly suppressed inflammatory response and oxidative stress. These benefits are likely mediated through inhibition of the NF-*κ*B signaling pathway.

To simulate the natural history of coronary heart disease, we developed a novel mouse model of MI superimposed on AS (11). Since atherosclerosis typically develops over many years before culminating in myocardial infarction, we implemented a 23-week high-fat diet prior to coronary artery ligation to thoroughly simulate disease progression. This extended period allowed the AS model mice adequate time to develop robust pathological adaptations and metabolic tolerance, thereby enabling the examination of artificial intervention and subsequent remodeling processes. This approach also aligns with the principles of primary prevention strategy evaluation.

The AS mouse model was established using Apoe^⁻/⁻^ mice fed a high-fat diet. Results demonstrated that AS model mice developed atherosclerotic lesions consistent with the pathological features of AS. The combination of AS and MI led to increased operative mortality and cardiac pathological changes characteristic of MI. Furthermore, assessment of NT-proBNP levels indicated post-MI cardiac functional alterations, with the AS + MI group exhibiting further functional deterioration. NT-proBNP is not only a marker of increased left ventricular wall stress but is also directly elevated in response to cardiac ischemia (18). Accordingly, cardiac function was more severely impaired in the AS + MI group than in the MI-only group, consistent with the observed pathological manifestations. Additionally, we investigated and compared the effects of AS and MI on inflammatory cytokine secretion. Our data indicated that Apoe^⁻/⁻^ mice in both the AS and AS + MI groups exhibited chronic inflammation by the end of the AS induction phase. Acute inflammation was further triggered following MI, indicating that MI exacerbates the inflammatory response. Results also revealed significantly enhanced oxidative stress in the AS + MI group. Through comprehensive cardiac ultrasound and histopathological examination, we demonstrated that AS exacerbates post-MI cardiac remodeling and dysfunction.

One of the most critical observations was the pronounced protective effect of HAL on cardiac structure and function after MI. The data indicated that AS significantly worsened MI-induced ventricular dilation, as reflected by increased LVIDd and LVIDs, and impaired systolic function, evidenced by reduced ejection fraction. HAL treatment markedly reversed these structural and functional deficits. The improvement in cardiac mechanics was consistent with changes in the serum heart failure biomarker NT-proBNP, which was highest in the AS + MI group and significantly lowered by HAL. Since ventricular remodeling is a pivotal process in post-MI heart failure progression, the ability of HAL to attenuate this remodeling highlights its potential therapeutic value. Importantly, this morphological and functional preservation translated into a substantial survival benefit, as HAL significantly reduced the high mortality rate associated with the AS + MI model.

Beyond these morphological and functional improvements, our findings uncover the fundamental pathological processes modulated by HAL. It has been established that inflammatory signals enhance adhesive interactions between leukocytes and endothelial cells, leading to the extravasation of neutrophils and monocytes. As infiltrating leukocytes clear necrotic cells from the infarct zone, mediators that suppress inflammation are released. The resolution of the inflammatory response is accompanied by the activation of reparative cells. Subsequently, fibroblasts proliferate, undergo transdifferentiation into myofibroblasts, and secrete substantial amounts of extracellular matrix proteins to preserve the structural integrity of the infarcted ventricle (19). The treatment produced marked anti-inflammatory effects, significantly reducing serum and cardiac levels of key pro-inflammatory cytokines (IL-1β, IL-6, TNF-α). While redox signaling tightly modulates the inflammatory response (20). Previous studies have shown that olive oil can improve ventricular remodeling after myocardial infarction by suppressing TNF-α and oxidative stress (21). The leaf extract and nuciferine prevented structural abnormality and inflammation in heart tissues after MI (9). Analogously, HAL addressed the oxidative stress component by decreasing oxidative damage markers (MDA, MPO) while enhancing antioxidant enzyme activities (SOD, GSH-Px). This dual modulation of inflammation and oxidative stress represents a critical intermediate mechanism through which HAL protects myocardial tissue from irreversible damage.

NF-κB was discovered 30 years ago as a rapidly inducible transcription factor. Since that time, it has been found to have a broad role in gene induction in diverse cellular responses, particularly throughout the immune system (22). NF-κB was discovered to play a central role at the nexus of persistent infections and chronic inflammation (23). Most significantly, our study identifies the inhibition of the NF-κB signaling pathway as the possiblemolecular mechanism underlying HAL's therapeutic effects. The reduction in phosphorylated p65 levels provides compelling evidence that HAL targets this master regulator of inflammatory response. By suppressing NF-κB activation, HAL effectively interrupts the inflammatory cascade at its source, thereby explaining its broad anti-inflammatory and subsequent cardioprotective actions. This mechanistic insight establishes a clear cause-effect relationship from molecular intervention to physiological outcome.

Furthermore, the inhibitory effect of HAL on atherosclerotic plaque formation, as shown by reduced lipid deposition in aortic tissue, suggests additional systemic benefits. By modulating lipid metabolism and promoting plaque stability, HAL may have created a more favorable systemic milieu, indirectly contributing to its cardioprotective effects after MI.

In summary, our findings indicate that HAL confers multi-faceted protection against post-MI remodeling and dysfunction, likely to pass throughby inhibiting the NF-κB pathway and its downstream inflammatory and oxidative effects. These results provide strong preclinical evidence supporting the further investigation of HAL as a promising therapeutic strategy for improving outcomes after myocardial infarction. Future studies should focus on elucidating its precise molecular targets and evaluating its long-term efficacy in different pathological settings.

## Data Availability

The original contributions presented in the study are included in the article/supplementary material, further inquiries can be directed to the corresponding authors.
